# A tetravalent nanovaccine that inhibits growth of HPV-associated head and neck carcinoma via dendritic and T cell activation

**DOI:** 10.1016/j.isci.2024.109439

**Published:** 2024-03-06

**Authors:** Romano Josi, Daniel E. Speiser, Simone de Brot, Anne-Cathrine Vogt, Eva M. Sevick-Muraca, Genrich V. Tolstonog, Martin F. Bachmann, Mona O. Mohsen

**Affiliations:** 1 Department of Rheumatology and Immunology, University Hospital of Bern, Bern, Switzerland; 2 Department of BioMedical Research, University of Bern, Bern, Switzerland; 3 Graduate School for Cellular and Biomedical Sciences (GCB), Bern, Switzerland; 4 Department of Oncology, Lausanne University Hospital and University of Lausanne, Lausanne, Switzerland; 5 COMPATH, Institute of Animal Pathology, University of Bern, Bern, Switzerland; 6 Center for Molecular Imaging, Brown Foundation Institute of Molecular Medicine, McGovern Medical School, The University of Texas Health Science Center at Houston, Houston, TX, USA; 7 Department of Otolaryngology – Head and Neck Surgery, Lausanne University Hospital and University of Lausanne, Lausanne, Switzerland; 8 Agora Cancer Research Centre, Lausanne, Switzerland; 9 Nuffield Department of Medicine, The Henry Welcome Building for Molecular Physiology, The Jenner Institute, University of Oxford, Oxford, UK; 10 Tajarub Research & Development, Doha, State of Qatar

**Keywords:** Immunology, Virology, Cancer

## Abstract

The global incidence of human papillomavirus (HPV) associated head and neck carcinoma is on the rise, in response to this a tetravalent therapeutic vaccine named Qβ-HPVag was developed. This vaccine, utilizing virus-like particles (VLPs) loaded with toll-like receptor ligands and chemically coupled to four HPV16-derived peptides, demonstrated strong anti-tumor effects in a murine head and neck cancer model. Qβ-HPVag impeded tumor progression, increased infiltration of HPV-specific T cells, and significantly improved survival. The vaccine`s efficacy was associated with immune repolarization in the tumor microenvironment, characterized by expanded activated dendritic cell subsets (cDC1, cDC2, DC3). Notably, mice responding to treatment exhibited a higher percentage of migratory DC3 cells expressing CCR7. These findings suggest promising prospects for optimized VLP-based vaccines in treating HPV-associated head and neck cancer.

## Highlights

• Qβ-HPVag impeded tumor progression in a murine head and neck model
• Qβ-HPVag augmented the infiltration of immune cells in the tumor microenvironment
• Therapeutic VLP vaccines are a promising strategy for HPV-related head and neck cancer

## Graphical abstract

**Figure:**
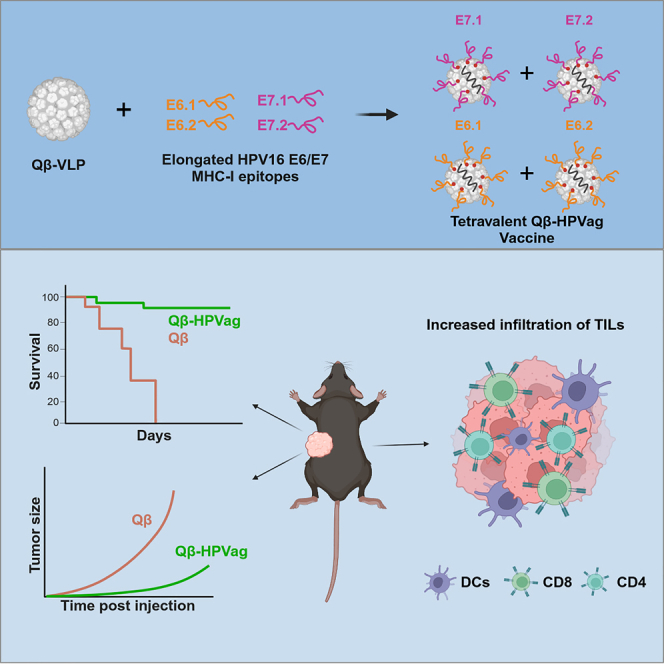

## Introduction

Head and neck cancer comprises a heterogeneous group of malignancies originating in the epithelial lining of the upper aerodigestive tract. The incidence of these cancers is on the rise globally, with a notable contributing factor being infection with human papillomavirus (HPV), particularly the high-risk subtype 16.^1^^,^^2^ HPV-associated oropharyngeal carcinoma is more prevalent in younger patients, and they tend to have markedly better outcomes following standard of care treatment when compared to patients with HPV-unrelated head and neck cancer.^3^ However, it is worth noting that conventional curative-intent therapies are associated with substantial morbidity.^4^ Furthermore, up to 36% of patients with HPV-associated head and neck cancer experience either locoregional relapse or distant metastasis within an eight-year timeframe.^5^^,^^6^ These challenges underscore the need for the development of more effective and less toxic immunotherapeutic approaches.

The objective of a therapeutic vaccine for HPV-associated head and neck cancer is to target and eliminate precancerous and cancerous lesions expressing the early oncogenic proteins E6 and E7.^7^^,^^8^ These proteins play a pivotal role in driving the proliferation of HPV-infected cells and continue to be expressed by the transformed cells.^1^ Consequently, they represent suitable targets for therapeutic vaccines.^3^ A range of approaches has been explored to develop and assess the anti-tumor response of therapeutic head and neck cancer vaccines, both in preclinical models and in clinical trials.^9^ Examples of therapeutic vaccines for HPV-associated head and neck cancer tested in clinical trials include: peptide-based vaccines^10^ (NCT02426892), an mRNA-based vaccine (NCT04534205), a DNA-based vaccine (NCT03162224), and a live-attenuated vaccine based on Listeria monocytogenes (NCT02002182).

Repetitive icosahedral virus-like particles (VLPs) represent a promising platform for the design and development of cancer vaccines. Their efficacy is attributed to several favorable characteristics, including safety, as these particles lack any replication competent viral genetic materials.^11^ The distinctive surface geometry of VLPs, representing a pathogen-associated structural pattern (PASP), further enhances their potential as vaccine candidates.^7^ Additionally, the ability to manipulate both the exterior and interior surfaces of VLPs as well as loading them with toll-like receptor (TLR) ligands provides a versatile framework for optimizing their immunogenicity and therapeutic potential in the context of cancer vaccination.^12^ VLPs have been specifically engineered for the development of effective therapeutic vaccines targeting HPV in preclinical models.^13^ Various studies have taken diverse approaches, including the design of T cell vaccines that target the oncogenic proteins E6 and E7, while others have concentrated on crafting chimeric vaccines that target both the capsid proteins and the oncogenic proteins.^14^^,^^15^^,^^16^^,^^17^

Dendritic cells (DCs) serve as critical antigen-presenting cells (APCs) responsible for bridging the innate and adaptive immune responses, and they play crucial roles in activating cytotoxic T cells. In fact, the effectiveness of cancer immunotherapy hinges on the interactions between DCs and CD8^+^ and CD4^+^ T cells, as well as other immune cells in the tumor microenvironment (TME). Increasing evidence confirms that DCs resident in tumors contribute to the regulation of T cell responses during immunotherapy.^18^^,^^19^^,^^20^^,^^21^^,^^22^ DCs are classified into distinct subsets, including cDC1 and cDC2, both originating from circulating precursors of conventional DCs (cDCs). cDC1s are particularly recognized for their pivotal role in cross-priming naive antigen-specific tumor CD8^+^ T cells within the tumor-draining lymph nodes (tdLNs) 23, 24. Indeed, studies have revealed that the extent of cDC1 infiltration in the TME is correlated with the efficacy of immunotherapy and prognosis in cancer patients.^23^ While cDC1s are specialized for activating CD8^+^ T cells, cDC2s exhibit functional and ontogenic heterogeneity. They are known to be essential for presenting exogenous antigens to CD4^+^ T cells and promoting the effective differentiation of T helper cells (T_H_ cells).^24^ Lastly, DC3s are likely derived from cDC1s or cDC2s and exhibit similarities to inflammatory DCs in inflamed tissues. However, the precise definition and nomenclature of DC subsets remain areas of ongoing research.^25^

The central objective of the present study is to provide a preclinical proof-of-concept for the therapeutic effectiveness of a tetravalent VLP-based vaccine loaded with a TLR 9-ligand (Qβ-HPVag). This vaccine is designed to present four distinct elongated epitopes derived from HPV16 E6 and E7. Additionally, the study aims to explore the impact of VLP-based vaccines on reshaping the TME, particularly on enhancing the presence of DCs and promoting the infiltration of antigen-specific T cells. There is still limited understanding regarding the impact of VLP-based vaccines on enhancing DC subsets infiltration in the tumor. This knowledge gap signifies an opportunity for ongoing and future research, offering the potential for valuable insights to optimize VLP-based vaccines.

## Results

### Development and characterization of a tetravalent nanovaccine (Qβ-HPVag) for targeted immunotherapy

**Figure 1:**
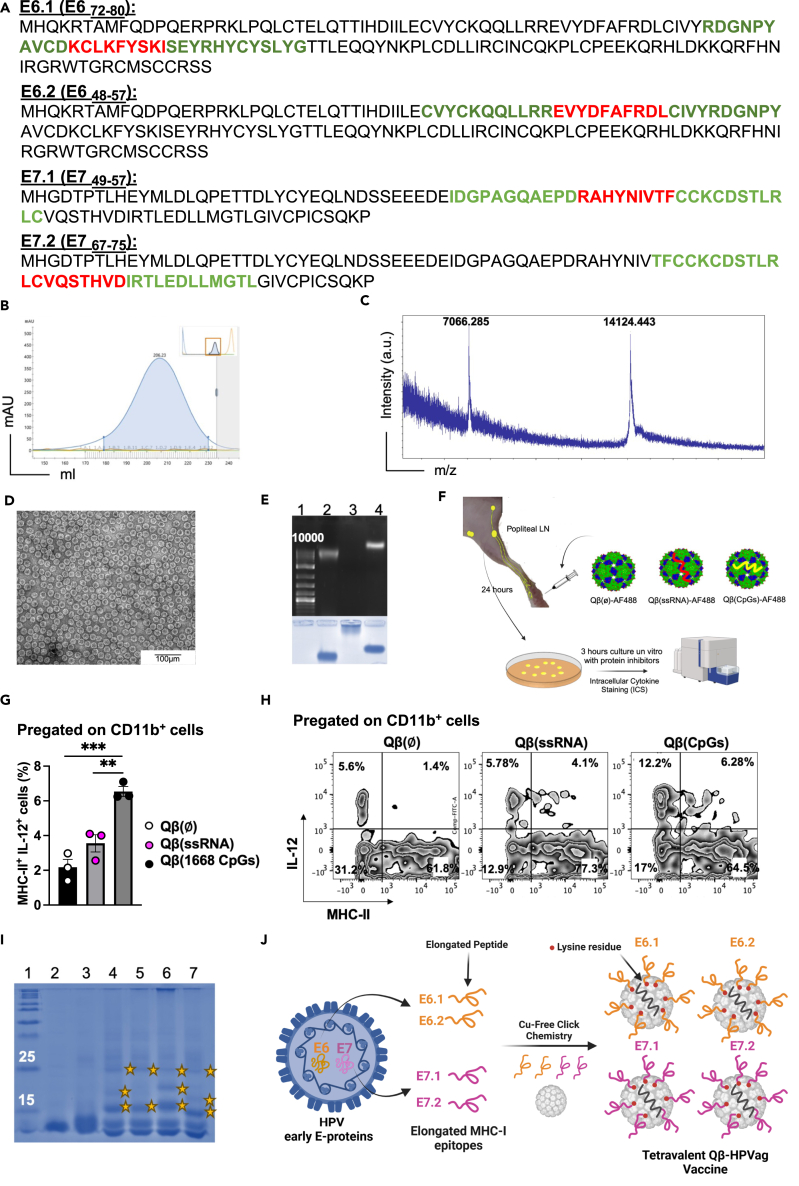
The production procedure of the tetravalent Qβ-HPVag (A) E6 and E7 selected oncogenic epitopes: (E6.1) E6_72-80_, (E6.2) E6_48-57_, (E7.1) E7_49-57_ and (E7.2) E7_67-75._ a.a. in red represents MHC-I epitopes and in green the extended flanking regions. (B) Fast Purification Liquid Chromatography (FPLC) ӒKTA pure chromatography system showing successful purification of Qβ-VLPs by Fractogel and Sephacryl S500 column, (mAu = milli-absorbance units). Briefly, Qβ nanoparticles are expressed in JM109 *E*. coli and then purified with a Fractogel and size exclusion columns. (C) Confirmation of successful expression of Qβ-VLPs was performed with mass spectrometry of purified Qβ-VLPs, the peak of 14124.443 Da corresponds to the Qβ monomer and the peak of 7066.285 Da corresponds to the Qβ capsid protein with two charges, m/z = mass/charge. (D) The integrity of the particles was confirmed with electron microscopy, images showed purified Qβ-VLPs, ∼30 nm in size. (E) 1% agarose gel stained with SybeSafe (Top): Lane 1: DNA marker, Lane 2: Qβ-VLPs spontaneously packaging ssRNA during the expression in *E*. *coli*. Lane 3: Qβ-VLPs Ø after successful enzymatic digestion of ssRNA, and Lane 4: Qβ-VLPs re-packaged with type B CpGs (Qβ(CpGs)). (Bottom) The same 1% agarose gel was stained with Coomassie Blue to confirm the integrity of the VLP’s protein capsid. (F) A cartoon illustrating the experimental plan of IL-12 detection. (G) Percentage of MHC-II^+^ IL-12^+^ cells, pregated on CD11b^+^ cells from the popliteal lymph nodes 24 h after subcutaneous injection with AF488 labeled: Qβ(Ø), Qβ(ssRNA) or Qβ(CpGs). (H) Representative FACS plot of MHC-II^+^ IL-12^+^ population in the various groups, pregated on CD11b^+^ cells. (I) 12% SDS-PAGE of the coupled elongated peptides to Qβ(CpGs) stained with Coomassie Blue, Lane 1: protein marker, Lane 2: Qβ(CpGs), Lane 3: Qβ(CpGs) derivatized with DBCO (Cu-free click chemistry) cross-linker, Lanes 4–7: Qβ(CpGs) coupled to peptides E6.1, E6.2, E7.1 and E7.2. Extra bands indicated with a star are peptide bound to a Qβ monomer. (J) A schematic representation illustrating the developmental process of the tetravalent virus-like particle (VLP)-based vaccine, denoted as Qβ-HPVag. Statistical analysis (mean ± SEM) by Student’s *t* test in G. The sample size was n = 3. Significance levels are denoted as follows: ∗∗∗∗p < 0.0001; ∗∗∗p < 0.001; ∗∗p < 0.01; ∗p < 0.05.

To design a murine VLP-based nanovaccine capable of displaying HPV16-derived E6 and E7 epitopes, we conducted an extensive review of published data that reported antigen-specific T cell responses observed in C57BL/6 mice. Our epitope selection process was guided by several key criteria: (1) *in vivo* anti-tumor efficacy in wild-type mice, as reported in studies^12^^,^^18^^,^^19^^,^^20^^,^^21^^,^^22^^,^^26^^,^^27^; (2) immunodominance; (3) peptide length, with a preference for 9–10 amino acids (a.a.); and (4) high binding affinity to major histocompatibility complex-I (MHC-I) molecules, as determined by NetMHCpan 4.1. Ultimately, we selected two peptides from E6 and two from E7 oncogenic proteins. The selected peptides were denoted as E6.1, E6.2, E7.1, and E7.2 (Figure 1A). A summary of these four chosen epitopes, along with their respective MHC-I binding strengths to H-2D^b^ and H-2K^b^, is provided in Table 1. E6.2 (E6_48-57_) and E7.1 (E_48-57_) likely cover immunodominant cytotoxic T lymphocyte (CTL) epitopes. The CTL epitopes in E6.1 and E6.2 have high binding affinity to H-2K^b^ molecules. The chosen E7.1 MHC-I epitope has high binding affinity to both H-2K^b^ and H-2K^d^ molecules. Despite not predicted to bind either H-2D^b^ or H-2K^b^ by the used prediction algorithm, E7.2 has been included as a fourth epitope due to favorable anti-tumor effects observed in preclinical HNC models.^28^^,^^29^

**Table 1:** Selected E6 and E7 epitopes and their binding to MHC-I

Antigen		MHC-I epitope	a.a.	H-2-Db (%rank)	H-2-Kb (%rank)	Reference
E6_72-80_	E6.1	KCLKFYSKI	9	3.9809	0.4193	Feltkamp, Peng et al.^28^^,^^30^
E6_48-57_	E6.2	EVYDFAFRDL	10	21.7904	0.8176	Feltkamp, de Oliveira et al.^28^^,^^31^
E7_49-57_	E7.1	RAHYNIVTF	9	0.01	1.0477	Feltkamp et al.^28^
E7_67-75_	E7.2	LCVQSTHVD	9	72.8846	89.8611	Feltkamp.Ferrantelli et al. et al.^28^^,^^29^

# Rank threshold for strong binding peptides 0.0500.

# Rank threshold for weak binding peptides 2.000.

Accessed NetMHCpan version 4.1, in Oct 2023.

To achieve effective immunotherapy, it is imperative to mobilize DCs because of their crucial role in regulating the immune response. To ensure effective stimulation of the innate immune system, we loaded our Qβ-VLPs (Figures 1B, 1C, and 1D) with type-B 1668 unmethylated CG-motif-containing oligonucleotides (CpG), a well-known ligand for TLR 9 (Figure 1E). To assess the efficacy of our nanoparticles in inducing an enhanced innate immune response compared to spontaneously packaged ssRNA-Qβ or the naked empty particle Qβ(Ø), we conducted the experiment outlined in Figure 1F. We measured the secretion of IL-12 in the popliteal lymph node (LN) 24 h after subcutaneous injection into the mouse footpad of either naked Qβ(Ø), Qβ(ssRNA), or Qβ packaged with oligonucleotides (Qβ(CpGs)). The results clearly demonstrated a significant increase in IL-12 secretion by activated CD11b^+^ DCs in the group that received Qβ(CpGs) when compared to those that received Qβ(ssRNA) (p = 0.0059) or the naked Qβ(Ø) (p = 0.0008) (Figures 1G and 1H).

Building upon former research,^32^ we have previously demonstrated that extending MHC-I epitopes by incorporating their natural flanking sequences can significantly enhance T cell activation and anti-tumor efficacy in comparison to shorter peptides.^33^ Accordingly, we designed peptides of approximately^30^ a.a. length (Figure 1A). The elongated peptides were synthesized with an additional lysine (K) a.a. and an azide group (N3) at the C-terminus as shown in (Table 2). The coupling of each peptide to Qβ was achieved through our well-established bio-orthogonal Cu-free click chemistry method using (dibenzocyclooctyne NHS ester DBCO linker). Briefly, the NHS ester group of DBCO reacts with the K residue on the surface of Qβ and incorporates cyclooctyne moiety which reacts—in a second step—with the (N3) labeled peptide to form a stable triazole linkage. The coupling was achieved through our well-established bio-orthogonal Cu-free click chemistry method, as outlined in the STAR methods section. Confirmation of the successful coupling of epitopes to Qβ(CpGs)-VLPs was obtained through SDS-PAGE analysis (Figure 1I). The resulting tetravalent nanovaccine was given the name Qβ-HPVag, as depicted in Figure 1J and the dosage was according to Table 3.

**Table 2:** Synthesized elongated peptides with natural flanking regions, additional lysine and an azide group (N3) at the C-terminus

Antigen	MHC-I epitope	Peptide elongation
E6.1	KCLKFYSKI	RDGNPYAVCDKCLKFYSKISEYRHYCYSLYGK(N3)
E6.2	EVYDFAFRDL	CVYCKQQLLRREVYDFAFRDLCIVYRDGNPYAK(N3)
E7.1	RAHYNIVTF	IDGPAGQAEPDRAHYNIVTFCCKCDSTLRLCK(N3)
E7.2	LCVQSTHVD	TFCCKCDSTLRLCVQSTHVDIRTLEDLLMGTL K(N3)

MHC-I epitope in red.

Flanking region in green.

Lysin (K) residue in blue.

Azide group (N3) in black.

**Table 3:** Groups, doses, and treatment schedule

Group	Dose	Treatment
Control	100μg	Days 3, 9 & 15 post-tumor inoculation
Vaccine	25μg E6.1 25μg E6.2 25μg E7.1 25μg E7.2 Total = 100μg	Days 3, 9 & 15 post-tumor inoculation

### The tetravalent Qβ-HPVag vaccine demonstrated significant anti-tumor efficacy *in vivo*

**Figure 2:**
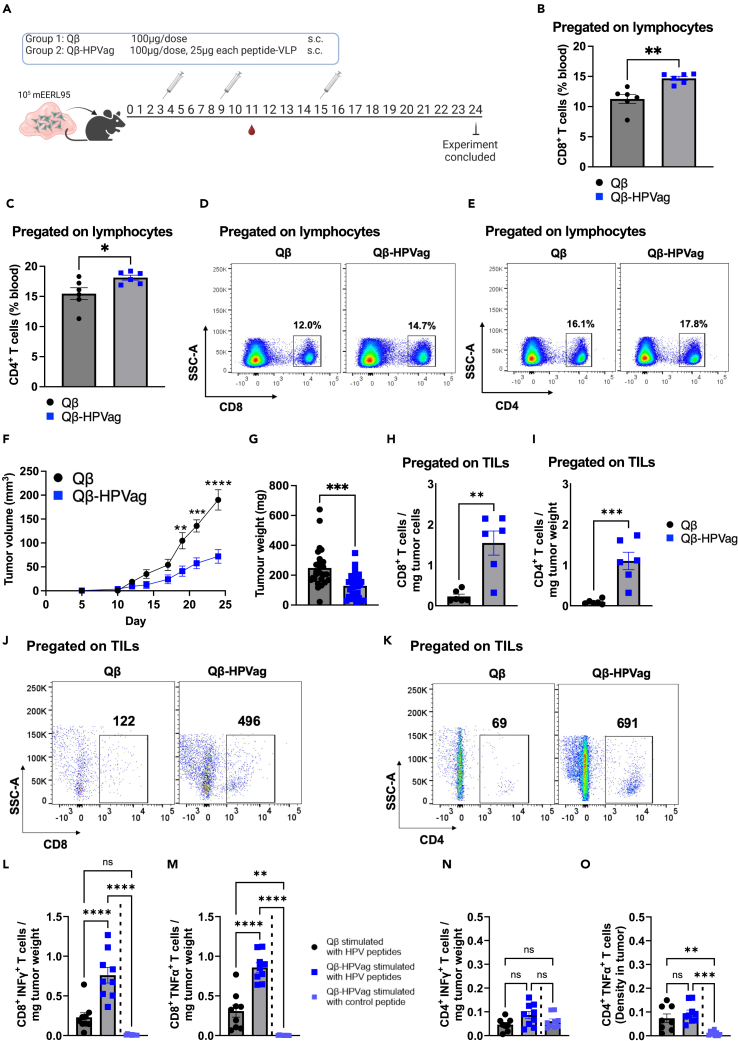
Anti-tumor efficacy of Qβ-HPVag (A) The experimental plan is depicted in the sketch. 10^5^ mEERL95 cells were formulated in Matrigel and inoculated subcutaneously in the mouse flank region. Two distinct groups were employed: the control group, represented by Qβ(CpGs) and the experimental group, which received the Qβ-HPVag vaccine. The treatment regimen involves the subcutaneous administration of a prime vaccination on day 3, followed by two subsequent booster vaccinations on days 9 and 15. Tumors were collected on day 24 and blood samples were collected on day 11. (B and C) Percentage of CD8^+^ and CD4^+^ T cells in the blood of mice on day 11, determined via flow cytometry (FACS) and analyzed in both groups. Each dot point on the plot represents an individual value. The gating strategy used involved a sequential selection of singlets, followed by lymphocytes, and further categorized into either CD8^+^ or CD4^+^ T cells. (D and E) Representative FACS plots illustrating the percentage of CD8^+^ or CD4^+^ T cells in blood in both groups. (F) Tumor size was evaluated by measuring tumor volume (mm^3^) in both groups. It’s worth noting that the control group reached the humane endpoint by day 24 due to the progression of tumor growth. (G) Tumor weight, measured in (mg), assessed on day 24 of the experiment, with each data point on the plot representing the weight of an individual tumor. (H and I) Densities of CD8^+^ and CD4^+^ T cells within the tumors. The densities were determined by calculating the ratio of the total number of the specific-cell population within each tumor to the tumor weight in (mg). (J and K) Representative FACS plots illustrating the total number of CD8^+^ or CD4^+^ T cells acquired from each tumor. The analysis was pregated on TILs. (L and M) Densities of CD8^+^ T cells producing IFN-γ^+^ or TNF-α^+^ within the tumor. (N and O) Densities of CD4^+^ T cells producing IFN-γ^+^ or TNF-α^+^ within the tumor. TILs isolated from mice vaccinated with Qβ-HPVag and stimulated with a control peptide are included (a separate experiment). The densities were determined by calculating the ratio of the total number of the specific-cell population within each tumor to the tumor weight in (mg). Statistical analysis (mean ± SEM) by Student’s *t* test in B, C, F, G, H and I. One-way ANOVA in L-O. The sample size was n = 6 in B, C, H, and I, n = 26 for Qβ and 29 for Qβ-HPVag group in G (2 experiments were combined), n = 9 in L-O. Significance levels are denoted as follows: ∗∗∗∗p < 0.0001; ∗∗∗p < 0.001; ∗∗p < 0.01; ∗p < 0.05.

In the next phase of our study, we evaluated the therapeutic anti-tumor efficacy of the tetravalent Qβ-HPVag in C57BL/6 mice bearing aggressive mEERL95 tumors. To do this, we subcutaneously inoculated 10^5^ mEERL95 tumor cells mixed with Matrigel into the flanks of the mice. Subsequently, the mice were vaccinated with either Qβ-HPVag or a control vaccine without peptide antigens (Qβ-(CpGs), referred to as Qβ), on days 3, 9, and 15, as shown in Figure 2A.

The administered doses were 100μg Qβ-HPVag (25μg of Qβ-E6.1, 25μg of Qβ-E6.2, 25μg of Qβ-E7.1 and 25μg of Qβ-E7.2) and the control (100μg of Qβ). We evaluated the systemic T cell responses eight days after the first immunization dose (day 11), which is typically the peak of the response. Our findings revealed a notable increase in both CD8^+^ and CD4^+^ T cell percentages in the group that received Qβ-HPVag compared to the control group which received Qβ (p = 0.0023 and p = 0.0324 respectively; Figures 2B–2E), suggesting induction of a peptide-specific response.

Tumor growth was notably delayed in the Qβ-HPVag vaccinated mice (p < 0.0001; Figures 2F and 2G). Our prior research has revealed a significant correlation between the density of CD8^+^ T cells within the TME and the resulting inhibition of tumor growth, leading to improved survival rates among the mice.^33^^,^^34^^,^^35^ To validate the effectiveness of our vaccination strategy, we assessed the tumor-infiltrating lymphocytes (TILs) on day 24 after inoculation of the mEERL95 tumor cell line. We measured the densities of CD8^+^ and CD4^+^ T cell populations within each tumor. The densities were determined by calculating the ratio of the total number of the respective cell populations within each tumor to the tumor weight in (mg). This approach allows for a standardized measurement of cell density that accounts for variation in tumor sizes. In the group that received the tetravalent Qβ-HPVag, we observed a noteworthy increase in CD8^+^ (Figures 2H and 2J) and CD4^+^ (Figures 2I and 2K) T cell densities when compared to the control group (p = 0.0014 and p = 0.0009, respectively). These findings strongly suggest that Qβ-HPVag has the capacity to enhance the infiltration of T cells and effectively restrain mEERL95 tumor growth.

TIL activity and functionality was assessed by harvesting tumors on day 24 following the administration of three doses of Qβ-HPVag or the control vaccine. TILs were isolated and stimulated with E6 and E7 or peptides. CD8^+^ T cells in the vaccinated group with Qβ-HPVag exhibited an augmented production of key cytokines, namely IFN-γ and TNF-α (Figures 2L and 2M). However, no significant increase in cytokine production was observed in CD4^+^ T cells within this group (Figures 2N and 2O). To gain a more comprehensive understanding of CD4^+^ T cell functionality and exhaustion markers at different stages of tumor growth following vaccination with Qβ-HPVag, future experiments should explore earlier time-points, aligning with the heightened T cell infiltration into the TME. This is an avenue that we are presently exploring.

### Augmented T cell infiltration and mitigated necrosis through Qβ-HPVag immunotherapy

**Figure 3:**
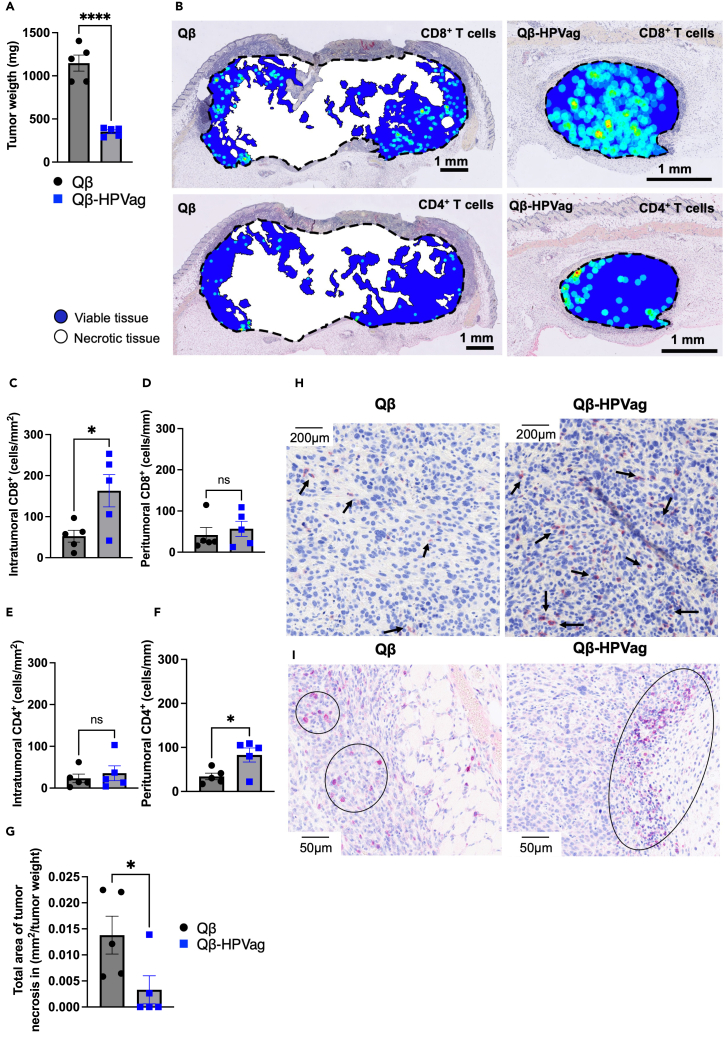
Histological assessment of mEERL95 tumors (A) The experimental plan is depicted in the sketch in Figure 2A. Tumor weight, measured in (mg), assessed on day 24 of the experiment, with each data point on the plot representing the weight of an individual tumor. (B) Visual colorization depicting CD8^+^ and CD4^+^ T cells in representative tumors for both control and vaccinated tissues is illustrated. The tumor border is delineated by a dashed black line, while areas of tumor necrosis are marked in white. Viable tumor regions are labeled in dark blue, featuring a visible heatmap determined by the count of CD8 or CD4 positive cells. Regions with the highest positive cell counts (‘hot’) are represented in red, while areas with the lowest positive cell counts are displayed in light blue (‘cold’). (C and D) Quantification of the number of intratumoral (expressed as cells/mm^2^) and peritumoral CD8^+^ T cells (expressed as cells/mm). (E and F) Quantification of the number of intratumoral (expressed as cells/mm^2^) and peritumoral CD4^+^ T cells (expressed as cells/mm). (G) The total area of tumor necrosis is measured in square millimeters (mm^2^), divided by the tumor weight, with each dot on the graph representing an individual tumor. (H) IHC staining of intratumoral CD8^+^ T cells in mice treated with either the Qβ control or Qβ-HPVag vaccine is depicted. CD8^+^ T cells are identified by black arrows (stained pink). (I) IHC staining of peritumoral (marginal) CD4^+^ T cells in mice treated with either the Qβ control or Qβ-HPVag vaccine is depicted. Circles highlight CD4^+^ T cell infiltration (stained pink). Statistical analysis (mean ± SEM) by Student’s *t* test in A, C, D, E, F and G. The sample size was n = 5. Significance levels are denoted as follows: ∗∗∗∗p < 0.0001; ∗∗∗p < 0.001; ∗∗p < 0.01; ∗p < 0.05.

We investigated T cell infiltration in the tumor and the adjacent tumor border, examining their distribution in viable and necrotic tumor regions through Hematoxylin and Eosin (H&E) and immunohistochemistry (IHC) staining. Employing a vaccination scheme according to Figure 2A, tumors were harvested on day 24. Consistent with earlier results, Qβ-HPVag demonstrated a hindrance to the progression of mEERL95 tumors (Figure 3A). Figure 3B shows a representative visual colorization of CD8^+^ and CD4^+^ T cells in a control tumor tissue (left) and the group vaccinated with Qβ-HPVag (right). In the control group (weighing 1404mg), CD4^+^ T cells exhibited overall low numbers, with some regional infiltration detected in the deepest marginal tumor areas, close to tumor necrosis. CD8^+^ T cells were uniformly distributed within the tumor, featuring small foci in various tumor regions. Contrastingly, in the vaccinated tumor with Qβ-HPVag (weighing 385.4 mg), CD4^+^ T cells were predominantly situated in the tumor margin (peritumoral area), with a distinct focal accumulation in the apical and lateral tumor region. CD8^+^ T cells demonstrated clear infiltration into the tumor, showcasing multifocal intratumoral foci concentrated in the lower portion of the tumor. Similar findings were obtained in other assessed tissues. In the subsequent phase, we conducted a quantitative analysis of intratumoral and peritumoral CD8^+^ and CD4^+^ T cells in both the control and Qβ-HPVag immunized groups, examining five tumors in each category. The intratumoral CD8^+^ T cell count exhibited a significant increase in the Qβ-HPVag group (p = 0.0295; Figures 3C and 3H). However, the difference of peritumoral CD8^+^ T cells only demonstrated borderline statistical significance (p = 0.05761; Figure 3D), possibly because many CD8^+^ T cells may have infiltrated the TME which would explain the observed anti-tumor response in the treated group with Qβ-HPVag. For CD4^+^ T cells, the peritumoral counts differed significantly between both groups (p = 0.0248; Figures 3F and 3I), while the intratumoral counts showed no significant disparity (Figure 3E). Afterward, we conducted a digital histological evaluation of the tumor tissues to measure the overall area of tumor necrosis. As previously demonstrated, tumor necrosis often attributed to hypoxia, indicates rapid cellular proliferation and correlates with cell proliferation in aggressive cancer.^34^^,^^36^ Our latest findings align with these earlier observations, revealing a notable reduction (p = 0.0491) in the tumor necrosis within the group treated with Qβ-HPVag (Figure 3G).

Collectively, these findings suggest the indispensable role of CD8^+^ T cells in instigating a potent anti-tumor response in the TME. The observed increased localization of CD4^+^ T cells at the tumor margin in the group receiving Qβ-HPVag might be attributed again to the specific time point at which these tumors were collected. An examination at an earlier time point could therefore also be informative following vaccination with Qβ-HPVag.

### The tetravalent Qβ-HPVag activates dendritic cells within the tumor microenvironment

**Figure 4:**
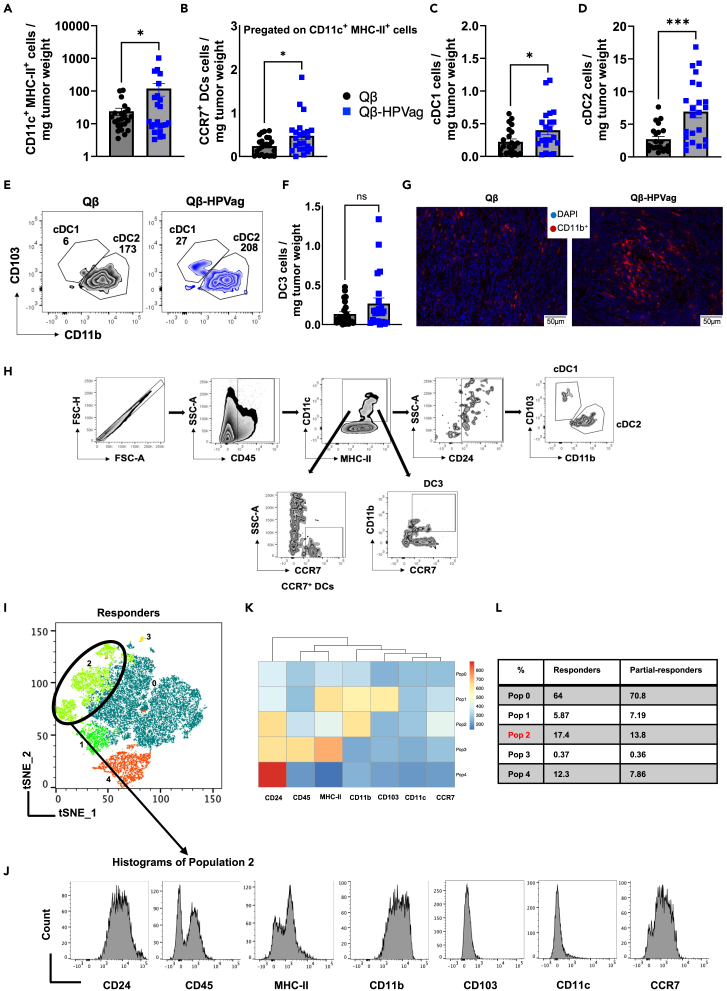
Dendritic cell subsets infiltrate mEERL95 tumors (A) Density of CD11c^+^ MHC-II^+^ cells in tumor, pregated on CD45^+^ cells. (B) Density of CCR7^+^ DCs, pregated on CD11c^+^ MHC-II^+^ cells in tumor. (C) Density of cDC1 cells in tumor (CD103^+^, CD11b^−^), pregated on CD45^+^, CD11c^+^/MHC-II^+^ and CD24^+^ cells. (D) Density of cDC2 (CD103^-^, CD11b^+^) cells in tumor, pregated on CD45^+^, CD11c^+^/MHC-II^+^ and CD24^+^ cells. (E) Representative FACS plots of cDC1 and cDC2 cells in tumors in the control and vaccinated groups. (F) Density of DC3 in tumor (CD11b^+^, CCR7^+^), pregated on CD45^+^, CD11c^+^/MHC-II^+^. (G) Immunohistochemistry of tumors in the control and the treated group, the cryosections were probed with antibodies detecting CD11b^+^ cells. (H) Detailed gating strategy for DC subsets (cDC1: CD11c^+^, MHC-II^+^, CD24^+^, CD11b^−^, CD103^+^; cDC2: CD11c^+^, MHC-II^+^, CD24^+^, CD11b^+^, CD103^-^; and DC3: CD11c^+^, MHC-II^+^, CD11b^+^, CCR7^+^). The densities were determined by calculating the ratio of the total number of the specific-cell population within each tumor to the tumor weight in (mg). (I–L) High-throughput analysis of the Qβ-HPvag vaccinated cohort, distinguishing responders and partial responders. (I) Concatenated tSNE clusters for DC subsets in the responders’ cohort, revealing five distinct clusters highlighted by unique colors for enhanced visualization. Markers employed include (CD45, CD24, CD11b, CD11c, CCR7, CD103, and MHC-II). (J) Histogram count for each marker used in population 2. (K) A heatmap presenting the expression of markers within each cluster. (L) A table comparing the percentage of each cluster in the responder and partial-responder cohorts. Statistical analysis (mean ± SEM) by Student’s *t* test in A, B, C, D, and F. The sample size was n = 23 in A, B and F, n = 22 in C and D, n = 8 in I-M. Significance levels are denoted as follows: ∗∗∗∗p < 0.0001; ∗∗∗p < 0.001; ∗∗p < 0.01; ∗p < 0.05.

To explore the presence of DCs within the TME subsequent to vaccination with Qβ-HPVag, we carried out additional experiments. Initially, we assessed the density of total CD11c^+^ MHC-II^+^ DCs in tumors from both vaccinated and control mice. The findings unveiled a noteworthy elevation in the density of activated DCs upon immunization with Qβ-HPVag (p = 0.0313; Figure 4A). The migration and localization of activated DCs within organs are orchestrated by the chemokine receptor 7 (CCR7).^37^^,^^38^ Accordingly, we evaluated the density of CCR7-expressing CD11c^+^ MHC-II^+^ cells in the tumor. Intriguingly, a notable increase was observed in the group that received the tetravalent Qβ-HPVag, underscoring the capacity of the vaccine to stimulate DCs within the TME (Figure 4B).

Subsequent to this, we characterized distinct DC subsets within the TME, specifically cDC1, cDC2, and DC3, employing the gating strategy delineated in Figure 4H (cDC1: CD11c^+^, MHC-II^+^, CD24^+^, CD11b^−^, CD103^+^; cDC2: CD11c^+^, MHC-II^+^, CD24^+^, CD11b^+^, CD103^-^; and DC3: CD11c^+^, MHC-II^+^, CD11b^+^, CCR7^+^). Our findings demonstrated a significant infiltration of both cDC1 and cDC2 within the tumors of Qβ-HPVag group (p = 0.0332 and p = 0.0003, respectively; Figures 4C–4E). While the DC3 population exhibited a noticeable upward trend following Qβ-HPVag administration, it is essential to highlight that this increase did not reach statistical significance (Figure 4F). Seeking more understanding of the presence of CD11b^+^ DCs within the TME in both the control and treated groups, we conducted immunofluorescent staining on tumor sections. Our results confirmed an escalation in the infiltration of Cb^+^ cells within the TME in Qβ-HPVag group (Figure 4G); CD11b^+^ serves as a prominent surface marker for both cDC2 and DC3 subsets (Figure 4H).

In the subsequent phase of our study, we conducted a more detailed analysis of the Qβ-HPVag treated group, dividing it into responders and partial-responders to gain deeper insights into the TME composition of DC subsets using the same panel of markers mentioned above (CD45, CD24, CD11b, CD11c, CCR7, CD103, and MHC-II). The 'responder' group was defined by tumors weighing a maximum of 100 mg. To ensure robust and consistent comparisons of unknown cell populations between these groups, we employed an unbiased automated analysis method, utilizing a non-linear dimensionality reduction technique, t-stochastic neighbor embedding (tSNE), followed by FlowSOM analysis, which revealed five different clusters (Populations 0, 1, 2, 3, and 4) of DC subsets within the TME following Qβ-HPVag treatment (Figure 4I). Notably, populations 1 and 3 displayed an approximately similar percentage in responders and partial-responders. The results indicated dissimilar percentage distributions among the other identified populations (0, 2, and 4). Population 0 exhibited an increase in the partial-responder group and demonstrated low expression of the utilized surface markers, underscoring the necessity for additional markers to investigate this population. Populations 2 and 4 exhibited considerably reduced size in the partial-responder group (Figure 4I). Population 2 exhibits the markers used to differentiate the DC3 subset and also shows overall increased expression of the migratory CCR7 receptor in the responder group. The generated tSNE heatmaps revealed increased expression of CD24 and CD11b and a slight expression of CD45, MHC-II, CCR7, and CD11c for population 2 (Figure 4K). Subsequently, we generated a histogram count analysis to validate these expression patterns in population 2 (Figure 4J). Population 4, reduced in the partial-responder group, displayed notably elevated expression solely in CD24, distinct from the other surface markers used. Overall, these findings indicate that DC subsets are critically affected in the TME following vaccination with Qβ-HPVag, which may play a pivotal role in elucidating the differences between responder and partial-responder groups.

### The tetravalent Qβ-HPVag reduced postsurgical tumor recurrence, which is dependent on the presence of CD8^+^ T cells

**Figure 5:**
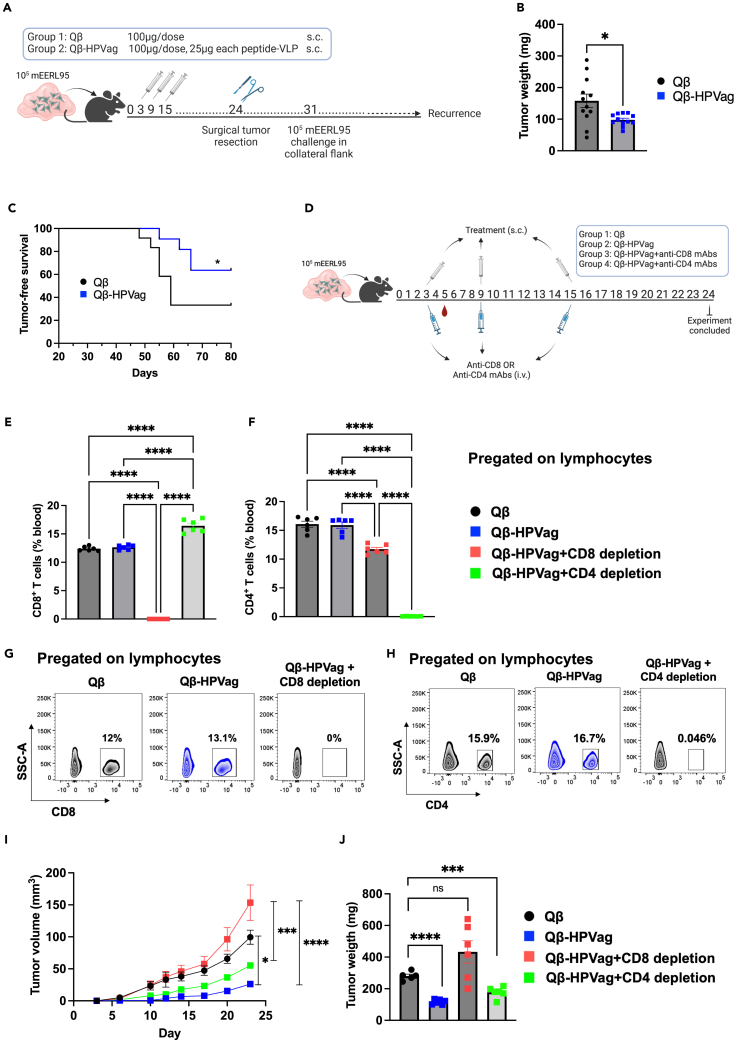
Qβ-HPVag enhance survival and anti-tumor activity is dependant on T cells (A) The experimental plan is depicted in the sketch. Initially, 10^5^ mEERL95 cells, combined with Matrigel, were subcutaneously inoculated in the mouse flank region. Subsequent vaccinations were administered on days 3, 9, and 15. Primary tumors were surgically removed on day 24 under isoflurane anesthesia. Afterward, mice were subjected to challenge by injecting 10^5^ mEERL95 cells combined with Matrigel subcutaneously into the opposite flank on day 31. The mice were then monitored daily for tumor growth (recurrence). Notably, no additional vaccinations were administered after the surgical resection of the primary tumors. (B) Tumor weight, measured in (mg), assessed on day 24 of the experiment, with each data point on the plot representing the weight of an individual tumor. (C) Tumor-free survival of the mice, with euthanasia being performed upon the detection of tumor relapse. (D) A sketch illustrating the experimental plan of the depletion experiment, anti-CD8 or anti-CD4 mAbs were administered intravenously on days 3, 9 and 15. Concurrently, treatment was administered subcutaneously on the same days. (E and F) Depletion efficiency, showing the percentage of CD8^+^ and CD4^+^ T cells in the blood of various groups. The gating strategy involved a sequential selection of singlets, followed by lymphocytes and further categorized into either CD8^+^ or CD4^+^ T cells. (G and H) Representative FACS plots for the data shown in panels E and F demonstrate the efficiency of T cell depletion in blood. (I) Tumor volume (mm^3^) measured over 24 days; each dot represents an individual tumor. (J) Primary dissected tumor weight, measured in (mg) on day 24 of the experiment. Statistical analysis (mean ± SEM) by Student’s *t* test in B, one-way ANOVA in E, F, I and J, log rank test for in C. The sample size was n = 12 in B and C, and n = 6 in E, F, I and J. Significance levels are denoted as follows: ∗∗∗∗p < 0.0001; ∗∗∗p < 0.001; ∗∗p < 0.01; ∗p < 0.05.

Encouraged by the observed anti-tumor efficacy of the Qβ-HPVag vaccine, we embarked on an investigation into its potential for preventing tumor relapse following the surgical resection of the primary tumor. To explore this, we designed a new experiment where a mixture of 10^5^ mEERL95 cells with Matrigel was subcutaneously inoculated followed by administration of a series of three vaccinations on days 3, 9 and 15. On day 24, we surgically removed the primary tumor as depicted in Figure 5A. One week after surgery, we subjected the mice to challenge with 10^5^ mEERL95 cells, which were subcutaneously inoculated into the collateral flank. We diligently monitored the mice for an additional 56 days to detect any signs of tumor regrowth, without administering additional vaccinations. As anticipated, Qβ-HPVag substantially curtailed tumor growth before resection (p = 0.0124; Figure 5B). Remarkably, Qβ-HPVag vaccination resulted in a significant improvement in survival, showing a 70% tumor-free survival in contrast to approximately 30% rate in the control group (p = 0.0375), all of this despite the absence of further immunizations (Figure 5C).

To investigate the influence of CD8^+^ and CD4^+^ T cells on the outcome of Qβ-HPVag immunization, we employed monoclonal antibodies (mAbs) to deplete these specific cell populations. This experiment encompassed four groups of mice: a control group treated with Qβ, a group receiving 3 tetravalent Qβ-HPVag injections over a 13-days period, and two additional groups (groups three and four) treated with the same Qβ-HPVag vaccine, in combination with either anti-CD8 or anti-CD4 monoclonal antibodies (Figure 5D). The depleting mAbs were administered intravenously on a weekly basis, ensuring an efficient depletion of CD8^+^ or CD4^+^ T cells, confirmed at levels of approximately 99–100% in the blood via flow cytometry (Figures 5E–5H). It is worth noting that the successful depletion of CD8^+^ T cells lead to a modest depletion of CD4^+^ T cells but not the other way round (Figure 5F). This effect occurred despite the use of low/few doses for depletion.

Consistent with our prior findings, Qβ-HPVag substantially inhibited tumor progression (p < 0.0001). Notably, the benefits of Qβ-HPVag vaccination were entirely abrogated when CD8^+^ T cells were depleted, leading to increased tumor growth and tumor weight, as observed on day 24 (Figures 5I and 5J). Unexpectedly, the depletion of CD4^+^ T cells only partially reduced the impact on the anti-tumor efficacy of Qβ-HPVag. This is consistent with the idea that CD4^+^ T cells promote the action of CD8^+^ T cells but show limited effector function themselves. In addition, the anti-CD4 mAb targets both conventional CD4^+^ T cells as well as T regulatory cells (Tregs), which will reduce tumor growth by itself. Taken together, these findings highlight the crucial role of CD8^+^ T cells in mediating the favorable anti-tumor effects of Qβ-HPVag vaccination.

## Discussion

In this study, we have designed a therapeutic tetravalent VLP-based vaccine (Qβ-HPVag) that displays elongated MHC-I epitopes derived from the E6 and E7 proteins of HPV16. The rationale behind incorporating elongated epitopes is grounded in both previous research by others and our own investigations. Accumulating evidence suggests that elongated epitopes exhibit superior anti-tumor efficacy compared to shorter ones. This observation holds true across various solid tumors, as evidenced by both preclinical and clinical studies as CD4^+^ T cells can deliver help to CD8^+^ T cells, in part by activating DCs through the CD40^−^CD40L signaling pathway.^32^^,^^33^^,^^39^^,^^40^ In the current preclinical research, E6 and E7 MHC-I murine epitopes have been specifically chosen. However, for humans, a broad range of human leukocyte antigens (HLAs) epitopes must be considered. Given the elongation of the MHC-I epitopes to approximately 32 a.a., it is anticipated that some sequences will also align with human HLAs. It is well-established that VLPs have an inherent capacity to provide additional T cell help beyond the primary benefits of the VLP itself, as they induce VLP-specific CD4^+^ T cells.^11^ Consequently, the activation of DCs through CD40L can also occur independently of tumor-specific T_H_ cells. Additionally, encapsulation of nucleic acids such as CpGs in VLPs induces DC activation, regardless of the presence of T_H_ cells.

The systemic increase in both CD8^+^ and CD4^+^ T cells in the blood stream after a prime and single boost administration of Qβ-HPVag indicates the efficacy of our vaccine construct. The increased T cell infiltration of both CD8^+^ and CD4^+^ T cells into the TME reinforces this observation. Indeed, assessing the density of TILs in the TME has been established in prior studies as a correlate of improved prognosis.^41^ It is important to note that measuring TILs density via flow cytometry lacks the precision to allocate these cells within the tumor environment. This precision can be achieved through standard HE staining and IHC. Notably, our data reveals a predominant allocation of CD8^+^ T cells within the tumor bed, with fewer cells at the margins, suggesting that a substantial proportion of CD8^+^ T cells may have infiltrated the TME which may explain the correlation to the induced anti-tumor response in the Qβ-HPVag group. This is further supported by the detrimental impact on tumor protection upon CD8^+^ T cell depletion. Contrastingly, when evaluating the localization of CD4^+^ T cells, opposite results emerge. Peritumoral CD4^+^ T cells were significantly higher in the Qβ-HPVag group whereas the intratumoral cells were not. We hypothesize that the time point post-treatment plays a role in the infiltration of CD4^+^ T cells into the tumor. In addition, the density of MHC class II expressing APCs at the tumor border may be higher than within the tumor, attracting the CD4^+^ T cells. Although cytokine production from CD4-positive T cells in the tumor was deemed insignificant 24 days post-tumor inoculation, CD8-positive T cells exhibited a notably increased IFN-γ and TNF-α production. The differences in cytokine production may also be attributed to the delayed assessment of CD4-specific T cell functionality. Future experiments should explore earlier time-points as well as different markers for effector function and exhaustion on CD4^+^ T cells.

Our study further revealed that therapeutic vaccination using the tetravalent Qβ-HPVag incorporating TLR 9 ligands leads to an elevated infiltration of diverse DC subsets within the TME. DCs in the Qβ-HPVag group exhibit heightened activation and migration capabilities compared to those in the control group. This observation is likely relevant, as DCs proficiently transport tumor antigens from the TME to the tumor draining LNs in a CCR7-dependent manner, facilitating the effective priming of anti-tumor CD8^+^ and CD4^+^ T cells.^37^^,^^42^^,^^43^ Notably, the cDC1 subset showed a significant increase in the Qβ-HPVag group, and the elevation in cDC2 was even more pronounced. Given that cDC2 can present exogenous antigens to CD4^+^ T cells and facilitate their differentiation, we hypothesize that peptide elongation may contribute to augmenting the infiltration of this specific subset into the TME. Interestingly, through high-throughput multi-parameter analysis, we discerned a notable differentiation between responder and partial-responder mice. Population 2 in both tSNE and FlowSOM analyses displayed distinct characteristics, mostly aligning with the DC3 population, marked by the CCR7 marker. Intriguingly, this specific population was reduced in partial-responder mice, suggesting that it is not only crucial to induce DC infiltration in the TME but also imperative for these cells to gain the ability of migrating to the tdLN through CCR7 expression. This migration is posited to be pivotal in eliciting more robust systemic protection against relapse, an area that is currently under investigation in our research endeavors. The reduction in Population 4 observed in the partial-responders group is noteworthy, particularly due to its high expression of the cell surface glycoprotein CD24. CD24, known for its diverse implications in cell adhesion, migration, and signal transduction, is expressed on various immune cells within the TME, such as T cells, B cells, macrophages, NK cells as well as on Tregs.^44^ The observed reduction in Population 4 suggests a potential impact of CD24-expressing cells on the response to vaccination with Qβ-HPVag. CD24’s involvement in immune cell interactions and modulation of immune responses may influence the dynamic changes within the TME following vaccination. Further profiling of this population may hold promise for providing deeper insights into how tumors in the responders' cohort behave post-vaccination with VLP-based vaccines.

Surgical resection is a key element in cancer management, but the risk of recurrence remains. Cancer vaccines, especially for high-risk patients with solid tumors like head and neck carcinoma, are promising options.^45^ Our data reveals a notable 70% tumor-free survival rate upon rechallenge in mice receiving the Qβ-HPVag vaccine, indicating potential activation of memory T cells. Remarkably, this effect is achieved without additional dosing. Exploring combination therapy with checkpoint inhibitors may be a strategy to further enhance the tetravalent Qβ-HPVag vaccine’s efficacy, potentially with synergistic effects for improving immune responses and outcomes in cancer treatment.

In conclusion, we demonstrated the efficacy of a preclinical tetravalent Qβ-HPVag vaccine in generating a potent anti-tumor response, preventing relapse in an aggressive murine head and neck cancer model. The vaccine enhanced T cell and DC subset infiltration in the TME, with a significant impact on local immune responses. Ongoing research aims to further optimize the vaccine’s efficacy and translate the findings for clinical application.

### Limitations of the study

The study uses a subcutaneous tumor flank model to study the effects of a cancer vaccine. While recognizing the advantages of this model, we acknowledge that it may not completely mimic the complex tumor microenvironment present in orthotopic settings.

## STAR★Methods

### Key resources table

**Table:** 

REAGENT or RESOURCE	SOURCE	IDENTIFIER
**Antibodies**		

CD16/CD32 anti-mouse	BD Biosciences	Cat# 553141 RRID: AB_394656
CD11b anti-mouse	ThermoFisher	Cat# 12-0112-82 RRID: AB_2734869
MHC Class II anti-mouse	ThermoFisher	Cat# 47-5321-80 RRID: AB_1548792
IL-12 anti-mouse	BD Biosciences	Cat# 56564 RRID: AB_1645243
CD8α anti-mouse	BD Biosciences	Cat# 552877 RRID: AB_394506
CD4 anti-mouse	BD Biosciences	Cat# 561832
CD45 anti-mouse	FisherScientific	Cat# 10140473
CD197 (CCR7) anti-mouse	ThermoFisher	Cat# 25-1971-82 RRID: AB_469652
CD11c anti-mouse	BioLegend	Cat# 117307 RRID: AB_3133776
MHCII anti-mouse	Abcam	Cat# ab242262
CD103 anti-mouse	BD Biosciences	Cat# 557494 RRID: AB_396731
CD24 anti-mouse	BD Biosciences	Cat# 562349 RRID: AB_1115896
CD11b anti-mouse	BioLegend	Cat# 101225 RRID: AB_830641
CD8α anti-mouse	BD Biosciences	Cat# 557654 RRID: AB-396769
INFγ anti-mouse	BD Biosciences	Cat# 554413 RRID: AB_398551
TNFα anti-mouse	BioLegend	Cat# 506321 RRID: AB_961435
InVivoMAb anti-mouse CD8β	BioXCell	Cat# BE0223 RRID: AB_2687706
InVivoMAb anti-mouse CD4	BioXCell	Cat# BE0003-1 RRID: AB_1107636
CD4 anti-mouse	ThermoFisher	Cat# 14-9766-82 RRID: AB-2573008
CD8 anti-mouse	ThermoFisher	Cat# 14-0808-82 RRID_2572861
GolgiPlug Protein transport inhibitor	BD Biosciences	Cat# 555029
Monensin	BioLegend	Cat# 420701

**Chemicals, peptides, and recombinant proteins**		

E6.1 HPV16 see Table 1	Pepscan	N/A
E6.2 HPV16 see Table 1	Pepscan	N/A
E7.1 HPV16 see Table 1	Pepscan	N/A
E7.2 HPV16 see Table 1	Pepscan	N/A
RNase A	Merck	Cat# 10109142001
ODN 1668 (CPG)	InvivoGen	Cat# tlrl-1668-5
Dibenzocyclooctin-N-hydroxysuccinimidylester (DBCO)	Merck	Cat# 761524-5mg
Matrigel	FisherScientific	Cat# 11573560

**Deposited data**		

Analyzed Data	This paper	N/A

**Experimental models: Cell lines**		

mEERL95	Department of Otolaryngology Lausanne University Hospital	N/A

**Experimental models: Organisms/strains**		

C57BL/6 mice	Inotiv	N/A

**Software and algorithms**		

GraphPad Prism V10.0	GraphPad Software, Boston, Massachusetts USA	N/A
Flowjo V10.10	BD Life Sciences	N/A
Visiopharm software	Visiopharm, HØrsolm, Denmark	N/A

### Resource availability

#### Lead contact

The experimental methods along with the data connected to this study can be obtained by contacting PD. Dr. Mona O. Mohsen (mona.mohsen@unibe.ch).

#### Materials availability

The study did not generate unique reagents.

#### Data and code availability

• The raw data can be requested from the lead contact upon request.
• This paper does not report any code.

### Experimental model and study participant details

#### *In vivo* experiments

Our experiments were conducted using seven to 11-week-old female C57BL/6 mice obtained from Inotiv in Amsterdam, Netherlands. The mice were accommodated at the central animal facility (CAF) of the Department of Biomedical Research (DBMR) at the University of Bern. All animal-related procedures and experiments were carried out in compliance with the regulations and under the approval of the veterinary authority of the Canton of Bern, Switzerland (BE43/21).

### Method detail

#### SHPV16 peptides

We selected the target T cell epitopes for HPV16 E6 and E7 oncoproteins based on previous literature. To predict peptide binding, we utilized NetMHCpan 4.1. We chose two epitopes from E6, denoted as E6.1 (HPV16 E6_72-80_, KCLKFYSKI) and E6.2 (HPV16 E6_48-57_, EVYDFAFRDL), and two E7 epitopes were named E7.1 (HPV16 E7_49-57_, RAHYNIVTF) and E7.2 (HPV16 E7_67-75_, LCVQSTHVD). We extended the MHC-I sequences to 32 a.a. using the natural flanking regions of the respective proteins. To facilitate coupling to Qβ-VLPs through bio-orthogonal Cu-free click chemistry, we added an additional lysine (K) and an azide group (N3) to the C-terminus of the peptide sequence. The additional (K) a.a. before the (N3) group is required to facilitate the synthesis of the peptide (a requirement for effective synthesis). The peptides used in this study were synthesized by Pepscan, and they were reconstituted in Dimethyle Sulfoxide (DMSO) from their lyophilized form. A detailed explanation of our established and used Cu-free click chemistry can be found in.^46^

#### Production and purification of Qβ-VLPs

Bacteriophage Qβ-VLPs were produced and purified as previously described.^33^ Briefly, the Qβ VLP expression plasmid containing the coat protein is transformed into an E.coli expression strain. Cells are harvested and the cell pellet is lysed. The Qβ VLPs are purified using a size-exclusion chromatography column. The integrity of the VLP is assessed by SDS-PAGE.

#### Production of the Qβ-HPVag vaccine

Qβ(ssRNA)-VLPs were used and the spontaneously packaged ssRNA was digested using RNase A (Merck). Successful digestion was confirmed with a 1% agarose gel. Qβ(Ø)-VLPs were repackaged with type B CpG (2.5μg/20μg Qβ-VLPs) (ODN1668, Invitrogen). The Qβ(Ø)-VLPs were mixed with CpG and incubated at 33°C for 3 h on a thermo-shaker. The repackaging was confirmed using 1% agarose gel. In a next step, Qβ(CpGs)-VLPs (in short “Qβ”) were derivatized for 1 h at room temperature using dibenzocyclooctyne NHS ester (DBCO bifunctional cross-linker, Sigma-Aldrich) in a 3-M excess. The reconstituted peptides were added in a 2-fold molar excess and incubated for 3 h at room temperature. The coupling to Qβ-VLPs for each peptide was performed separately. Successful peptide coupling to Qβ-VLPs was confirmed using a 12% SDS-PAGE gel. Please note that all Qβ-VLPs in this paper are Qβ(CpGs) as ssRNA was always replaced by type B 1668 CpGs.

#### IL-12 detection

Seven to 11-week-old female C57BL/6J mice were subcutaneously immunized in the footpad with 50μg of Qβ(Ø) or Qβ(ssRNA) or Qβ(CpGs) under anesthesia. Popliteal lymph nodes were collected after 24 h and single-cell suspension was prepared and cultured for 3 h with GolgiPlug (BD Biosciences, 1:1000) and Monensin (BioLegend, 1:1000). Cells were stained with FcR-block antibody (CD16/CD32, clone 2.4G2, BD Biosciences) for 15 min at 4°C. The following cell surface anti-mouse fluorophore mAbs were used: CD11b (PE, clone M1/70, eBioscience), MHC-II (I-A/I-E, APC-Cy7, clone M5/114.15.2). Intracellular cytokine staining was performed as described later for IL-12 (p40/p70, FITC, clone 15.6, BD Pharmingen). Samples were read with a BD LSRII and analyzed using Flojo(V.10) and GraphPad Prism (V10).

#### Tumor cell line

mEERL95 cell line was kindly provided by Genrich Tolstonog^47^ (University of Lausanne, Lausanne, Switzerland). The mEERL95 was cultured in Gibco Dulbecco`s Modified Eagle Medium (DMEM) F-12 medium supplemented with 10% fetal bovine serum (FBS), 1% penicillin/streptomycin and 1x human keratinocyte growth supplement (HKGS). When cells6reached 80% confluency the growth media was aspirated, and cells were washed three times with 1x phosphate buffered saline (PBS). After washing, 1x trypsin was added to the T75 Flask and incubated at 37°C and 5% CO_2_ for 7 min. Cells were either resuspended in a growth medium for continued culture or in 1x PBS admixed with Matrigel (Corning) for cell injection. The cell line was tested several times for Mycoplasma infection using Microsart AMP Mycoplasma Kit.

#### Tumor model and treatment schedule

The mEERL95 tumor cells were resuspended in a mixture of 30 μL of PBS and 20 μL of Matrigel (Corning). Subsequently, 10^5^ tumor cells were subcutaneously injected into the left flank of each mouse. The specific doses for both the control and the tetravalent vaccine can be found in Table 3. Tumor size was assessed every other day using a caliper, and the tumor volume was calculated using the formula 0.5 × (length × width^2^). To adhere to ethical guidelines, mice were euthanized before the tumor reached a humane-end point volume of 1 cm^3^, in accordance with the animal license (BE43/21). After 24 days, the experiment was concluded, and the tumors were excised for further analysis. The doses mentioned in Table 3 represent the concentrations of VLPs, either in isolation (control) or in conjunction with various peptides.

#### Single-cell isolation from peripheral blood samples

Blood samples were obtained from the tail vein of tumor-bearing mice and collected in Eppendorf tubes containing 0.5M EDTA (Fluka, Biochemika). To isolate white blood cells, red blood cells (RBCs) were lysed using ACK-Buffer (0.155M ammonium chloride, 0.01M potassium hydrogen carbonate, pH 7.2). Following a 5–7 min incubation on ice with ACK buffer, cells were resuspended in FACS buffer (PBS +2% FBS) within 96-well U-bottom plates. For optimal staining, cells were pre-incubated with FcR-block (CD16/CD32, clone 2.4G2, BD Biosciences) for 15 min at 4°C. Subsequently, cells were stained with anti-CD8α (PE-Cy7, clone 53–6.7, BD Biosciences) and anti-CD4 (PE, clone H129.19, BD Biosciences) for 20 min at 4°C. After staining, the cells underwent three washes and were resuspended in 400μL of FACS-buffer. The gating strategy on lymphocytes is illustrated in Figure S1A.

#### Single-cell isolation of tumor-infiltrating lymphocytes (TILs)

Single-cell suspensions were prepared as follows. Briefly, 24 days post mEERL95 cell inoculation, tumors were surgically dissected, weighed, and processed for subsequent analysis. Tumors underwent a 45-min incubation with Collagenase D 1 mg/ml (Roche) at 37°C. The resulting cell suspension was mechanically passed through a 70μm cell strainer (Sigma-Aldrich). Subsequently, cells were layered onto a 35% Percoll gradient (Sigma-Aldrich) to enhance the purification of TILs from stromal cells. When necessary, red blood cells (RBCs) were lysed using ACK-Buffer, as previously described. The isolated TILs were then resuspended in FACS-Buffer within a 96-well U-bottom plate. For optimal staining, single-cell suspensions were treated with FcR-Block (CD16/CD32, clone 2.4G2, BD Biosciences) for 15 min at 4°C, followed by centrifugation for 5 min at 300g. CD8^+^ and CD4^+^ cell quantification employed anti-mouse fluorophore monoclonal antibodies (mAbs): CD8α (PE-Cy7, clone 53–6.7, BD Biosciences) and CD4 (PE, clone H129.19, BD Biosciences). For DC characterization, the following anti-mouse fluorophore mAbs were utilized: CD45 (PE-Texas red, clone RA3-6B2, BD Biosciences), CCR7 (CD197, PE-Cy7, clone 4B12, eBioscience), CD11c (PE, clone N418, Biolegend), MHC-II (VioletFluor 450, clone M5/114.15.2, Abcam), CD103 (FITC, clone M290, BD Biosciences), CD24 (APC, clone M1/69, BD Biosciences), and CD11b (APC-Cy7, clone M1/70, BioLegend). Staining was conducted for 30 min at 4°C in the dark. After surface staining, cells were washed three times and resuspended in 400μL FACS-buffer. Sample analysis was performed using a BD LSRII and evaluated with Flojo (V.10) and GraphPad Prism (V10). The gating strategy on TILs is illustrated in Figure S1B.

#### Intracellular cytokine staining

For intracellular cytokine staining (ICS), TILs were resuspended in DMEM (+10% FBS, 1% PenStrep) and transferred to 96-well plates. A mixture containing 100 U/ml of mouse interleukin-2 (mIL-2, Roche) and 1 μg/mL of each peptide was added, followed by incubation for 24 h at 37°C. On the subsequent day, the media was changed, and the stimulation cocktail was mixed with GolgiPlug (BD Biosciences, 1:1000) and Monensin (BioLegend, 1:1000). After another 16 h of incubation at 37°C, cells were washed three times using DMEM medium and resuspended in FACS Buffer. For optimal staining, single-cell suspensions were treated with FcR-Block (CD16/CD32, clone 2.4G2, BD Biosciences) for 15 min at 4°C, followed by centrifugation for 5 min at 300g. Subsequently, cell surface anti-mouse fluorophore monoclonal antibodies (mAbs) CD8 (APC-Cy7, clone 53–6.7, BD Biosciences) and CD4 (PE, clone H129.19, BD Biosciences) were used for 30 min at 4°C in the dark. After surface staining, cells were washed once with FACS buffer and then fixed with cytofix buffer (BD Biosciences) for 15 min at 4°C. Following fixation, cells were washed with FACS buffer and permeabilized using 1× perm buffer (Invitrogen). Subsequently, cells were centrifuged at 300g for 5 min and treated with the intracellular staining cocktail, which included anti-mouse fluorophore mAbs IFN-γ (APC, clone XMG1.2, BD Biosciences) and TNF-α (Percep-Cy5.5, clone MP6-XT22, BioLegend) resuspended in 1x perm buffer, and incubated for 20 min at 4°C. After intracellular staining, cells underwent three washes and were resuspended in 400 μL of FACS buffer. Samples were analyzed using a BD LSRII, and data analysis was performed using Flojo (V.10) and GraphPad Prism (V10). In an additional experiment, mice were vaccinated with the vaccine Qβ-HPVag and tumors were collected for TILs isolation. A similar protocol as described above was performed and the TILs were stimulated with a cocktail containing 1 μg/ml of an elongated control peptide derived from the 4T1 mammary carcinoma cell line (CFSAFGNRKNLKYNAVPTVFAFQNPTEVCPEK(N3)),^33^ GolgiPlug (BD Biosciences, 1:1000) and Monensin (BioLegend, 1:1000).

#### Immunofluorescence of tumor sections

Tumors were carefully excised with adequate margin from the mice on day 24 as illustrated in Figure 2A, embedded in Tissue-Tek OCT compound (Sakura, 4583), and promptly frozen on dry ice. The OCT-embedded tumors were then sectioned into 12μm slices using a cryostat. These sections were fixed with ice-cold pure acetone for 10 min and air-dried for an additional 15 min. To ensure optimal preparation, the sections were prewetted three times in PBS for 5 min each. Subsequently, slides were blocked with 1% BSA and 1% normal mouse serum in PBS for 1 h. Following the blocking step, the sections were directly stained with a CD11b antibody (CD11b, APC, Clone M1/70, BD Biosciences) diluted to a concentration of 1:500 in the blocking buffer. Staining was carried out for 1 h, followed by three washes with PBS for 5 min each. Finally, sections were mounted with a single drop of Fluoroshield with Dapi (Merck, F6057), and images were captured using a Zeiss Axio Imager.A2 microscope.

#### High dimensional analysis of flow cytometry data

High dimensional analysis of flow cytometry data was performed using t-distributed stochastic neighbor embedding (tSNE) and FlowSOM clustering and visualizing technique. FCS files of TILs were used following compensation and gating on single cells and live cells using FlowJo X version 10.4.2. FCS files (8 FCS files from each group) were exported and then concatenated; each used file included a consistent number of events (5000 events), total concatenated events was 40,000. Five clusters were applied as mentioned in the figure legends. The resulting subpopulations were further analyzed for specific marker expression (CD45, CD24, CD11b, CD11c, CCR7, CD103 and MHC-II). In FlowSOM analysis, five clusters were applied, as indicated in the figure legends. The resulting subpopulations were then subjected to further analysis to assess specific marker expression using histogram count.

#### T cell depletion

In experiments involving the depletion of CD8^+^ or CD4^+^ cells, mice received intravenous injections of either 50μg of anti-CD8β antibodies (clone 53–5.8, BioXcell) or 50μg of anti-CD4 antibodies (clone GK1.5, BioXcell) on a weekly basis. The efficiency of depletion was evaluated by assessing peripheral blood samples 48 h after the initial depletion. Tumor growth was assessed using the method described above.

#### Survival experiments

In the survival experiment, the primary tumor was surgically removed under anesthesia 24 days after inoculation with the cell line. The wound closure was performed using a 9mm autoclip applier (Clay Adams 9mm autoclip applicator, Vet-Tech). Subsequently, mice received an antidote mixture (Revertor 0.22 mg/mL, Anexate 0.09 mg/mL, Temgesic 0.015 mg/mL) in accordance with our BE43/21 license. Following the surgery, mice were allowed one week to recover, after which they were subcutaneously re-challenged in the collateral flank with 10^5^ mEERL95 cells in Matrigel. Euthanasia of animals occurred upon the observation of local recurrence or collateral tumor growth in accordance with the criteria outlined in our BE43/21 license.

#### Histology

Formalin-fixed tumors collected on day 24 as illustrated in Figure 2A were used for histology, n = 5 from each group. Tumor tissue was histologically examined by a board-certified veterinary pathologist (SdB). Of each tumor, a complete cross section was trimmed and routinely processed for histologic evaluation. Tissue sections were stained for hematoxylin and eosin (HE) as well as for CD4 (clone 4SM95, rat, Thermo F. Scientific, 14–9766) and CD8 (clone 4SM15, rat, Thermo F. Scientific, 14-0808-82) immunohistochemistry. Both antibodies were diluted 1:200. Detection and quantification of tumor tissue, tumor necrosis, and CD4^+^ and CD8^+^ cells was performed digitally using Visiopharm software (Visiopharm, Hørsholm, Denmark). The number of CD4^+^ and CD8^+^ cells was calculated per mm (tumor periphery) and per mm^2^ (intratumoral area). Based on CD4^+^ and CD8^+^ cell counts, visual colorization were generated in order to determine the spatial distribution of T cells.

### Quantification and statistical analysis

Data are expressed as mean ± SEM. Analyses were performed using GraphPad Prism V10. Comparisons between two groups were conducted using Student’s *t* test (two-tailed), while one-way ANOVA was employed for the analysis of more than two groups. Tumor growth curves were assessed using either Student’s *t* test or one-way ANOVA. Tumor-free survival was analyzed utilizing the log rank (Mantel-Cox) test. Significance levels are denoted as follows: ∗∗∗∗p < 0.0001; ∗∗∗p < 0.001; ∗∗p < 0.01; ∗p < 0.05. Refer to the figure legends for detailed information regarding the performed analyses.

## Supplemental information

Figure S1. The gating strategy for lymphocytes (A) Gating strategy for single cells followed by lymphocytes in blood. (B) Gating strategy for single cells followed by lymphocytes in TILs.
