# Supplementary material for: A tetravalent nanovaccine that inhibits growth of HPV-associated head and neck carcinoma via dendritic and T cell activation
Source: iScience. 2024 Mar 6;27(4):109439. doi: 10.1016/j.isci.2024.109439 (PMC10957412; doi:10.1016/j.isci.2024.109439)
Supplement: Figure S1. The gating strategy for lymphocytes — (A) Gating strategy for single cells followed by lymphocytes in blood. (B) Gating strategy for single cells followed by lymphocytes in TILs. [file mmc1.pdf]

iScience, Volume *27*

Supplemental information

A tetravalent nanovaccine that inhibits growth of HPV-associated head and neck carcinoma via dendritic and T cell activation

Romano Josi, Daniel E. Speiser, Simone de Brot, Anne-Cathrine Vogt, Eva M. Sevick-Muraca, Genrich V. Tolstonog, Martin F. Bachmann, and Mona O. Mohsen

A)

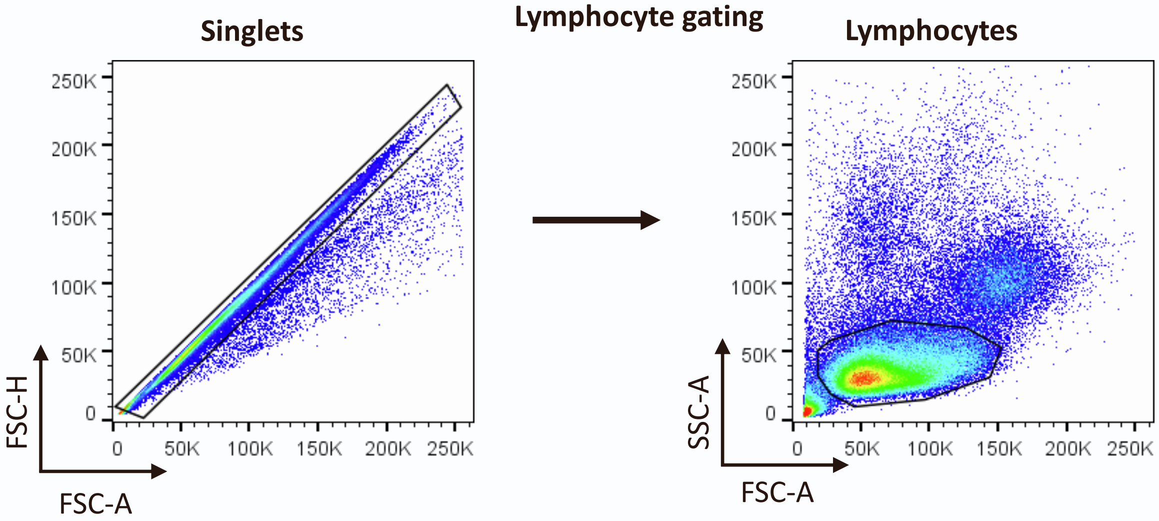

Lymphocyte gating
Singlets
Lymphocytes
FSC-H
FSC-A
SSC-A
FSC-A
0
50K
100K
150K
200K
250K

B)

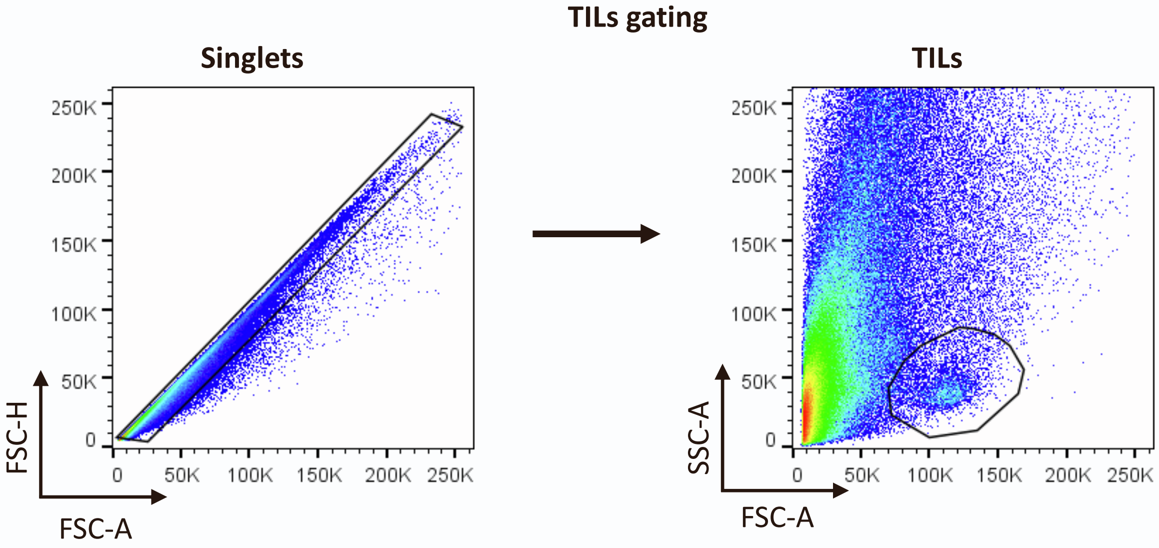

TILs gating
Singlets
TILs
FSC-H
FSC-A
SSC-A
FSC-A
0
50K
100K
150K
200K
250K
